# Editorial: Advancements in P2X7 antagonists: from mechanistic insights to therapeutic applications

**DOI:** 10.3389/fphar.2026.1912316

**Published:** 2026-07-14

**Authors:** Natiele Carla da Silva Ferreira, Rômulo José Soares Bezerra, Rodrigo da Cunha Bisaggio

**Affiliations:** 1 Laboratory of Cellular Communication, Oswaldo Cruz Institute, Oswaldo Cruz Foundation, Rio de Janeiro, Brazil; 2 Laboratory of Technological Development in Virology, Oswaldo Cruz Institute, Oswaldo Cruz Foundation, Rio de Janeiro, Brazil; 3 Federal Institute of Education, Science and Technology of Rio de Janeiro, Rio de Janeiro, Brazil

**Keywords:** drug development, inflammatory conditions, neurological disorders, P2X7 antagonists, pain, purinergic receptors

The purinergic receptor P2X7, cloned in the 1990s, is distinctive in that, beyond the initial opening of a cationic channel at lower ATP concentrations, sustained stimulation leads to the formation of a membrane pore permeable to molecules up to 900 Da (Alves et al., 2014). P2X7 receptor activation triggers pro-inflammatory pathways that are implicated in neurodegeneration, chronic inflammation, and pain, which affect millions of people worldwide (da Silva Ferreira et al., 2019). Although several antagonists have been developed and tested in clinical trials (e.g., ADZ9056 from AstraZeneca), none have achieved regulatory approval, highlighting the urgent need for innovative therapeutic strategies and the discovery of novel molecules. The Research Topic “*Advancements in P2X7 antagonists: from mechanistic insights to therapeutic applications*” presents six articles summarizing recent progress in elucidating the mechanisms of P2X7 antagonism and their translational potential.

Liu et al. reviewed the contributions of the P2X7 receptor to neurodegenerative diseases. In Alzheimer’s disease, Parkinson’s disease, multiple sclerosis, and amyotrophic lateral sclerosis, P2X7 receptor activation promotes neuroinflammation. This process involves the activation of microglia, the release of inflammatory cytokines via the NLRP3 inflammasome, the production of reactive oxygen species and nitric oxide, which drive oxidative stress and neurotoxicity, and an increase in intracellular calcium, which contributes to apoptosis. Blocking the P2X7 receptor reduces neuroinflammation, microglial activation and cognitive deficits in mice. The receptor also influences microglial polarization between M1 (pro-inflammatory and damaging) and M2 (anti-inflammatory and reparative) profiles, with imbalances contributing to disease progression. These findings highlight new perspectives for the development of therapeutic strategies for neurodegenerative disorders.

Both Zhang et al. and Jiang et al. reviewed the role of the P2X7 receptor in pain. Microglia are the primary cells expressing P2X7 receptor in the Central Nervous System, where its activation promotes an M1 profile associated with pain states. This process involves the release of IL-1β, which contributes to central sensitization, as well as other mediators such as tumor necrosis factor-α, inducible nitric oxide synthase, prostaglandin E2, cyclooxygenase-2, and brain-derived neurotrophic factor. Conversely, knockdown strategies or pharmacological blockades can shift microglia toward an M2 profile, alleviating the pain. The P2X7 receptor may also facilitate signaling between glial cells and neurons under elevated ATP levels. Both studies emphasize evidence supporting analgesia through P2X7 receptor inhibition or knockdown, underscoring the need for continued research into novel antagonists as potential therapeutic approaches.

In the field of cardiovascular diseases, Liu et al. demonstrated the role of the P2X7 receptor in aggravating ischemic stroke. During a stroke, neuronal death leads to the release of large amounts of ATP into the extracellular space, which enhances the inflammatory response through P2X7 receptor activation. Infiltrating CD4^+^ T cells, such as Th1 and Th17 cells, further exacerbate neurological damage by releasing cytokines, injuring neurons, activating glial cells, and disrupting the blood–brain barrier. The P2X7 receptor can also activate T cells, promote their proliferation via nuclear factor of activated T-cells/IL-2, and induce naïve T cell polarization toward Th1/Th17 cells. The authors observed increased P2X7 receptor expression in infiltrating CD4^+^ T cells in the brains of mice after stroke. Conversely, P2X7 knockout mice exhibited smaller ischemic volumes, improved neurological performance on the rotarod test, reduced neuronal apoptosis, and lower levels of IL-17A, IL-21, and phosphorylated STAT3. These findings indicate that the P2X7 receptor drives Th17 differentiation via the IL-21/STAT3 pathway, worsening injury. Thus, inhibiting this receptor may provide a therapeutic strategy to mitigate ischemia–reperfusion damage.

Tan et al. explored the role of the P2X7 receptor in chronic kidney disease, showing that activation of the NLRP3 inflammasome via the P2X7 may contribute to structural alterations and increased tissue inflammation. They reported elevated expression of both the receptor and NLRP3, as well as higher extracellular ATP concentrations in animals subjected to unilateral ureteral obstruction. End-stage renal disease is often characterized by renal interstitial fibrosis, with collagen deposition and α-smooth muscle actin (α-SMA) expression contributing to extracellular matrix accumulation, myofibroblast activation, and progressive tissue scarring in the kidneys. The authors demonstrated that blocking the P2X7 receptor with the antagonist BBG reduced both collagen accumulation in the tubulointerstitial region and α-SMA expression. These findings suggest that the P2X7 receptor could be a promising therapeutic target for the treatment of renal interstitial fibrosis.

In the field of drug discovery, Lima et al. conducted a comparative study of structural models of the human P2X7 receptor generated using different modeling approaches. For years, homology modeling has been the conventional method for protein structure prediction. However, in the late 2010s, AlphaFold revolutionized the landscape of structural biology by winning both the 13th (CASP13) and 14th (CASP14) Critical Assessment of Structure Prediction competitions. These achievements marked a turning point in protein science and set the stage for a new era of drug discovery (Abbass, 2026). In 2024, Demis Hassabis and John Jumper of Google DeepMind, the creators of AlphaFold, were awarded the Nobel Prize in Chemistry for their groundbreaking contributions to protein structure prediction (The Nobel Prize, 2024). Despite these advances, no prior study has assessed the relevance and accuracy of both approaches for the P2X7 receptor and their application in virtual screening. The authors conducted a comparative analysis of two versions of AlphaFold (AlphaFold2 and AlphaFold3) against homology modeling using the SWISS-MODEL. The results showed that all three approaches were effective in structural prediction and in reproducing interactions with key binding-site residues, with AlphaFold2 demonstrating notable accuracy and consistency. These findings demonstrate the applicability of AlphaFold-based models in virtual screening, potentially accelerating the search for new P2X7 receptor antagonists and inspiring research on other members of the P2X receptor family.

Together, these studies illustrate how mechanistic insights are being translated into clinical opportunities. With advances in molecular modeling and immunology, the P2X7 receptor is emerging as a compelling therapeutic target for neurodegeneration, renal disease, pain, and other inflammatory conditions, driving the search for novel molecules and paving the way for clinical validation.
